# Antitumor Compounds From Halophilic *Streptomyces violaceorubidus* M4 Against Triple‐Negative Breast Cancer

**DOI:** 10.1002/mbo3.70095

**Published:** 2025-10-27

**Authors:** Atousa Zia, Ensieh Salehghamari, Hanieh Jalali, Maryam Taher

**Affiliations:** ^1^ Department of molecular medicine Pavia University Pavia Italy; ^2^ Department of Cell and Molecular Biology, Faculty of Biology Kosar University of Bojnord Bojnord Iran; ^3^ Department of Animal Biology, Faculty of Biological Sciences Kharazmi University Karaj Iran

**Keywords:** apoptosis, *CASPASE 8* gene, *P53* gene, *Streptomyces violaceorubidus*, triple‐negative breast cancer

## Abstract

Triple‐negative breast cancer (TNBC) is an aggressive form of breast cancer characterized by the absence of estrogen and progesterone receptors and minimal HER2 expression, restricting the available treatment options. Actinobacteria have emerged as promising sources of anticancer compounds because of their remarkable ability to produce beneficial compounds. This study aimed to evaluate the antitumor effects of the halophilic *Streptomyces violaceorubidus* M4 extract on TNBC both in vitro and in vivo. The extracted compounds were analyzed by LC‐MS. MTT and annexin‐PI assays were used to assess the apoptosis‐inducing effects of the compounds on MDA‐MB‐231 and MCF‐10A cells. The expression of apoptosis‐related *BAX*, *BCL2*, *P53, CASPASE‐8*, and *CASPASE‐9* genes was analyzed using Real‐time PCR. A TNBC mouse model was established using 4T1 cell transplantation, and the animals received the extract intravenously for 21 days. *S. violaceorubidus* M4 contained bioactive compounds, including amino acids, carboxylic acids, coumarins, isoflavones, phosphatidylcholine, tetrahydroxyanthraquinone, and flavonoids. The extract demonstrated selective cytotoxicity against MDA‐MB‐231 cells, with an IC50 of 48.04 μg/mL after 48 h, while the IC50 for MCF‐10A cells was 132 μg/mL. The reduction in *Cas‐9* expression alongside the elevation of *Cas‐8* and *P53* expression suggests the participation of the extrinsic pathway in the process of apoptosis. Histopathological evaluation of tumor tissues from mouse models showed that the extract injection reduced the number of mitotic cells, nuclear pleomorphism, and angiogenesis in tumor tissue. This study suggests that *S. violaceorubidus* M4 has a pronounced anticancer effect on TNBC and can be considered for the production of anticancer substances.

## Graphical abstract

The antitumor effects of the halophilic *Streptomyces violaceorubidus* M4 extract on triple‐negative breast cancer, highlighting its selective cytotoxicity and apoptosis‐inducing properties. Key findings include the identification of bioactive compounds and their impact on apoptosis‐related gene expression, as well as significant reductions in tumor cell proliferation in a mouse model.

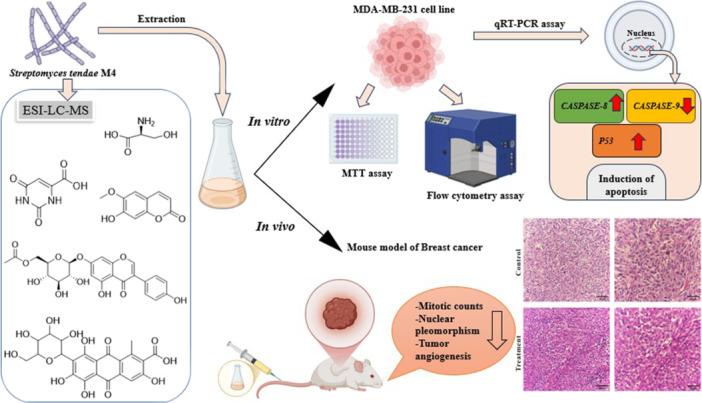

## 1 Introduction

Breast cancer is the most prevalent cancer and the leading cause of mortality among women (Li and Kwon 2019). In 2022, around 2.3 million new cases were reported globally, resulting in 666,000 deaths. Globally, breast cancer accounts for 23.8% of all cancer diagnoses and 15.4% of cancer‐related deaths in women (Zhang et al. 2025). Breast cancer classification depends on immunohistochemical staining to identify the presence of three crucial receptors: estrogen receptor (ER), progesterone receptor (PR), and human epidermal growth factor‐2 (HER2). Triple‐negative breast cancer (TNBC) is a particularly aggressive form of breast cancer, representing 15%–20% of breast cancer cases, and lacks specific targeted therapies (Zagami and Carey 2022). TNBC is defined as HER2‐negative with less than 1% expression of estrogen and progesterone receptors. For early‐stage TNBC, standard treatment protocols primarily involve surgical intervention followed by postsurgical adjuvant chemotherapy to prevent cancer recurrence (Cardoso et al. 2019). Remarkable progress in the survival rates of HR‐positive and HER2‐positive breast cancers has been made possible by the development of cutting‐edge treatments, such as monoclonal antibodies targeting HER2, Antibody‐drug conjugates, and inhibitors of cyclin‐dependent kinase 4/6 (CDK4/6) (Martin et al. 2022; von Minckwitz et al. 2019). TNBC has limited treatment options because it does not respond to effective therapeutic agents, such as hormone therapies and anti‐HER2 treatments, leading to the worst prognosis among all breast cancer types. TNBC may manifest as invasive ductal carcinoma or tissue‐specific types, such as cystic, adenoid, and secretory types (Zagami and Carey 2022; Geyer et al. 2017). Disease recurrence after surgery and treatment is a significant challenge in breast cancer management (Valachis et al. 2018).

Natural compounds have gained increasing importance as potential cancer treatments owing to their favorable safety profiles, fewer side effects, and capacity to address resistance to chemotherapeutic drugs (Naeem et al. 2022). These compounds also enhance antiproliferative effects and effectively target a range of signaling pathways in various cancer types, including TNBCs (Malla et al. 2020). Actinomycetes are gram‐positive bacteria distinguished by a high guanine‐cytosine (G: C) ratio in their genomic architecture. Some actinomycetes, known as halophiles, exhibit a remarkable ability to adapt to extreme environments, such as saline soils, making them appealing for scientific research because of their novel bioactive compounds with various applications in human health (Valan Arasu et al. 2016). Recent research indicates that these halophilic bacteria may play a significant role in the prevention and treatment of cancer because of their capacity to synthesize anticancer compounds (Pongen et al. 2023; Corral et al. 2019; Mariadhas Valan et al. 2016). The antitumor agents under discussion are divided into several structural categories, including anthracyclines, enediynes, indolocarbazoles, isoprenoids, macrolides, and nonribosomal peptides. These substances exhibit antitumor activity mainly by triggering apoptosis through mechanisms such as DNA cleavage, which is facilitated by the inhibition of topoisomerase I or II, mitochondrial permeabilization, and the inhibition of key enzymes involved in signal transduction, such as proteases, and cellular metabolism. Furthermore, some compounds may exert their effects by preventing tumor‐induced angiogenesis (Olano et al. 2009).

Numerous studies have shown that actinomycete‐derived extracts possess cytotoxic properties against a range of cancer cell lines (Zhang et al. 2017). Notably, extracts from *Streptomyces* species exhibit considerable cytotoxicity towards lung cancer (A549) and breast cancer (MCF‐7) cell lines (Wang et al. 2022; Flores Clavo et al. 2021). These actinomycete extracts can initiate apoptosis in cancer cells through several pathways, including caspase activation and alteration of B‐cell lymphoma‐2 (Bcl‐2) family proteins. Moreover, some actinomycete extracts have been found to cause cell cycle arrest at specific stages, thereby preventing the proliferation of cancer cells. For example, certain extracts from *Streptomyces* can halt the cell cycle at the G2/M phase (Kouroshnia et al. 2022).

Given the increasing incidence of breast cancer, it is essential to identify novel, effective, and readily accessible pharmacological agents for its treatment. These new drugs should be effective against resistant tumors while reducing side effects and enhancing treatment outcomes. In light of this need, we investigated the antitumor potential of a recently isolated strain of halophilic *Streptomyces violaceorubidus* M4. This study focused on the effects of this compound on the MDA‐MB‐231 breast cancer cell line, which is characterized as triple‐negative, as well as on a mouse model of TNBC.

## 2 Materials and Methods

### 2.1 Preparation of Ethyl Acetate Extract From the Actinobacterial Strain

*Streptomyces* sp. M4, a halophilic actinomycete, was obtained from the microbiology laboratory at Kharazmi University in Tehran, Iran, and cultured according to a standardized protocol (Salehghamari et al. 2022). Briefly, strain M4 was incubated in starch casein broth (SC agar) containing soluble starch (20 g/L), K_2_HPO_4_ (0.5 g/L), KNO_3_ (1 g/L), MgSO_4_·7H_2_O (0.5 g/L), and NaCl (30 g/L) at 28°C and 220 rpm for 4 days. For metabolite extraction, ethyl acetate was used as the solvent in a 1:2 v/v ratio for 2 h. The Ethyl acetate layer, which potentially contains cytotoxic metabolites, was subsequently evaporated using a rotary evaporator set at 40°C.

### 2.2 Genome Extraction and Molecular Characterization of *Streptomyces* Sp. M4

Genomic DNA was isolated from strain M4 using a Bacterial DNA Isolation Kit (Kiagene Fanaver, Iran) according to the manufacturer's instructions. The 16S ribosomal DNA (rDNA) segment from the isolated DNA was then amplified through polymerase chain reaction (PCR) using the primers 9F (5‐AAG AGT TTG ATC ATG GCT CAG 3') and 1542R (5‐AGG AGG TGA TCC AAC CGC 3'). PCR was performed in a 20 µL reaction volume using a T100 Thermal Cycler (Bio‐Rad, Hercules, CA, USA). The reaction mixture included 1× PCR Buffer, 0.2 mM dNTPs, forward and reverse primers each at a concentration of 0.5 pmol/μL, Taq enzyme at 0.065 U/μL, 5 µL of template DNA, 1.5 mM MgCl_2_ solution, and 9.15 µL of double‐distilled water. The thermal cycling conditions were as follows: initial denaturation at 94°C for 5 min, followed by 30 cycles of denaturation at 94°C for 30 s, annealing at 57°C for 30 s, and extension at 72°C for 60 s. After completing the cycles, the reaction was maintained at 72°C for 10 min before being cooled to 4°C. The PCR products were identified using 1% gel electrophoresis and subsequently analyzed by Macrogen Inc. (Seoul, South Korea). The EzTaxon server (http://www.ezbiocloud.net/eztaxon) was used to determine the phylogenetic relationships among the isolates and to compute pairwise 16S rDNA sequence similarities. The sequences obtained, along with reference sequences from GenBank, were aligned using the multiple‐sequence alignment software CLUSTAL X version 1.81 Phylogenetic.

### 2.3 LC‐MS Analysis of *Streptomyces* Sp. M4 Extract

To identify the bioactive compounds in the extract, LC‐MS analysis was conducted using a Micromass Quattro micro‐API mass spectrometer (USA) paired with a Waters Alliance 2695 high‐performance liquid chromatography (HPLC) system (USA). The mobile phase comprised 0.1% formic acid in acetonitrile (Channel A) and 0.1% formic acid in deionized water (Channel B). Chromatographic separation was performed on a Eurospher C18 column with dimensions of 4.6 × 120 mm, operating at a flow rate of 0.3 mL/min. The LC conditions were set to 10% A for the first 0–10 min, shifting to 50% A from 8 to 15 min, and reaching 80% A from 15 to 30 min. The analytical method used a flow rate of 3.0 mL/min, an injection volume of 5 μL, and a column temperature of 35°C. The mass spectrometer functioned in the positive ion mode, with a mass detection range of 100–1400 atomic mass units (amu), and a capillary voltage of 4000 V. Analyte ionization was achieved using a chemical ionization source under the following conditions: source temperature, 120°C; desolvation temperature, 300°C; and gas flow rate, 300 L/h.

### 2.4 Cellular Maintenance and Growth Conditions

The MDA‐MB‐231 and MCF‐10a cell lines were obtained from the Pasteur Institute of Iran and cultured in DMEM/F‐12 medium (Bioidea, Iran). For MCF‐10a cells, a specialized culture medium was prepared by combining DMEM/F‐12 with additional components: 20 ng/mL EGF, 5% horse serum, 100 ng/mL cholera toxin, 250 ng/μL hydrocortisone, and 10 μg/mL insulin. The culture medium was supplemented with 10% (v/v) heat‐inactivated fetal bovine serum (FBS; Gibco, Brazil), along with streptomycin (100 ng/mL) and penicillin (100 IU/mL) from Bioidea, Iran. The cells were maintained at 37°C in an environment with 90% humidity and 5% CO_2_. Upon reaching 80% confluence, the cells were transferred to a fresh medium for continued growth.

### 2.5 Cytotoxicity Assay

Cells were seeded at a density of 1 × 10^4^ cells/well in a 96‐well plate and allowed to adhere for overnight. The following day, the cells were exposed to 0–500 μg/mL of extract for 24, 48, and 72 h. Following treatment, 10 μL of MTT solution (5 mg/mL, Sigma Company, USA) was added to each well and mixed thoroughly. After incubation for 4 h, the supernatants were extracted, and 100 μL of DMSO was added to each well to solubilize the precipitates. Cell viability was assessed by measuring the absorbance at 570 nm using an ELISA plate reader (Bio‐Rad, Hercules, CA, USA). The proportion of viable cells was determined by comparing the absorbance of the extract‐treated cells with that of the untreated controls, and the results were expressed as a percentage of the control.

### 2.6 Dual Staining With Annexin‐FITC and Propidium Iodide

An annexin V‐FITC apoptosis detection kit (MabTag, Germany) was used to assess the apoptosis. The MDA‐MB‐231 cell line was plated in six‐well plates at a concentration of 1 × 10^6^ cells/mL and incubated for 24 h. Following this, the cells were treated with 48.08 μg/mL extract for 48 h. Posttreatment, the cells were harvested via trypsinization and resuspended in 100 μL of binding buffer. The cell suspension was then incubated with 1 μL of annexin V‐FITC and propidium iodide (PI) for 10 min at room temperature in the dark. A BD FACSLyric flow cytometer (US) was used to measure the annexin V‐positive cell population. The percentage of apoptotic cells (annexin V+) was calculated using FLOWJO software (FlowJo Engine v4.00770).

### 2.7 RNA Isolation and Quantitative Real‐Time Polymerase Chain Reaction (qRT‐PCR)

The cells were grown in six‐well plates until they reached approximately 80% confluence. The extract was then applied to the cells at a concentration of 48.08 μg/mL. Following a 48‐h incubation, RNA was extracted using the RNX‐Plus RNA isolation kit (Cinna Gen Co, Tehran, Iran) according to the manufacturer's guidelines. cDNA was synthesized using a high‐capacity cDNA reverse transcription kit (Parstous, Iran) according to the manufacturer's instructions. qRT‐PCR was conducted using PowerUp SYBR Green master mix (Thermo Scientific), utilizing 10 ng of cDNA as the template. Beta‐2‐microglobulin (B2M) was used as the reference for normalizing gene expression, and the 2^−ΔΔCt^ method was used to determine fold changes. The expression levels of BAX, BCL2, P53, CASPASE 8, and CASPASE 9 were examined using the primers listed in Table 1. This analysis incorporated gene expression data from three separate biological experiments.

**TABLE 1:** List of primer pairs used for qRT‐PCR amplification.

Gene	Sequence	Product size (bp)
*B2M*‐F	5'‐ GACCACTTACGTTCATTGACTCC ‐ 3'	171
*B2M*‐R	5'‐ CAGGGTTTCATCATACAGCCAT ‐ 3'	
*BAX*‐F	5'‐TCAGGATGCGTCCACCAAGAAG ‐ 3'	103
*BAX*‐R	5'‐TGTGTCCACGGCGGCAATCATC ‐ 3'	
*BCL2‐F*	5'‐GGTGGGGTCATGTGTGTGG ‐ 3'	89
*BCL2‐R*	5'‐CGGTTCAGGTACTCAGTCATCC ‐ 3'	
*P53‐F*	5'‐CCTCAGCATCTTATCCGAGTGG‐ 3'	128
*P53‐R*	5'‐TGGATGGTGGTACAGTCAGAGC‐ 3'	
*CASPASE 8*‐F	5'‐ AGAAGAGGGTCATCCTGGGAGA ‐ 3'	142
*CASPASE 8*‐R	5'‐ TCAGGACTTCCTTCAAGGCTGC ‐ 3'	
*CASPASE 9*‐F	5'‐GTTTGAGGACCTTCGACCAGCT ‐ 3'	129
*CASPASE 9*‐R	5'‐CAACGTACCAGGAGCCACTCTT ‐ 3'	

### 2.8 Animals and Tumor Induction

Six‐to 8‐week‐old female BALB/c mice were obtained from the Pasteur Institute of Iran. These mice were housed in conditions with a constant temperature range of 22°–24°C and experienced 12‐h cycles of light and darkness, with unrestricted access to food and water. This study adhered to the ARRIVE guidelines and was approved by the Ethics Committee of Tehran University (IR.UT.SPORT.REC.1403.065). Following a week‐long adaptation period, six female mice were randomly assigned to two groups using the RAND function of Microsoft Excel. The control group (*n* = 3) was administered 100 μL of normal saline, whereas the experimental group (*n* = 3) was injected with 1 × 10^6^ 4T1 cells to develop a breast cancer model. Twenty‐two days after tumor establishment, 10 mg/kg actinomycete extract in 100 μL of water for injection was administered to the experimental group via the tail vein every other day for 21 days. The mice were monitored daily, and their body weight and tumor size were measured. After the treatment period, the animals were euthanized using sodium thiopental (40 mg/kg). Tumor tissues were removed from each mouse for histopathological analysis. Tumor volume was calculated using the following formula (Kawasaki and Sendo 2021):

$$\text{Tumor volume}=\frac{1}{2}\times \text{Length}\times {\text{Width}}^{2}.$$

### 2.9 Histological Examination

Tumor tissues were stored in 10% buffered formalin. Subsequently, the samples were dehydrated using a series of alcohol concentrations (100, 95, 90, 80, 70, and 50%). The treated tumor tissues were subsequently embedded in paraffin blocks and sliced into 5 μm thick sections. The sections were then deparaffinized, rehydrated, stained with hematoxylin and eosin, and observed under a light microscope (Layton and Suvarna 2013; Aboubakr et al. 2020).

### 2.10 Statistical Analysis

Data analysis was performed using GraphPad Prism software (version 10.3.1). One‐way ANOVA was used for MTT assay analysis, followed by the least significant difference test for multiple comparisons. For Real‐time PCR and histopathological analyses, a non‐parametric *t*‐test was applied. Results are presented as mean ± SEM, with statistical significance set at *p* < 0.05.

## 3 Results

### 3.1 Molecular Characterization of the Actinomycete Strain and Its Metabolites

**FIGURE 1:**
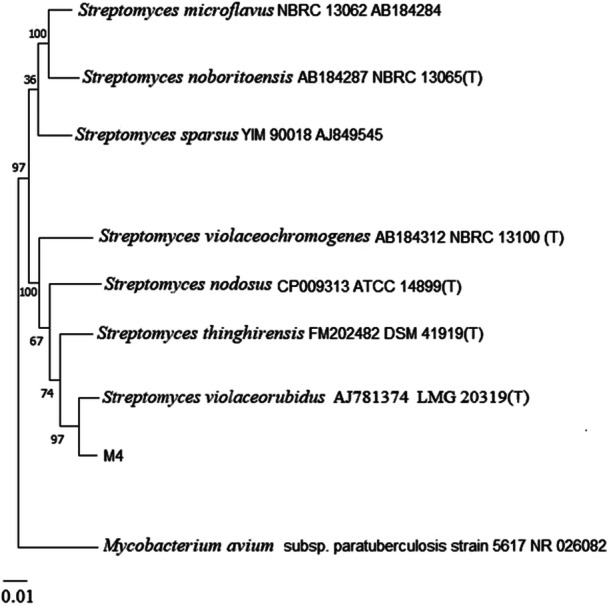
A neighbor‐joining tree using partial 16S rDNA sequences illustrated the relationship between strain M4 and the genus *Streptomyces*, with *M. avium* as the outgroup. Bootstrap values from 1000 resamplings are indicated at the branch nodes, and the 0.01 bar represents substitutions per nucleotide position. T: Type strain.

Partial 16S rDNA gene sequence of strain M4 demonstrated a 100% similarity to *S. violaceorubidus* LMG 20319. A partial 16S rDNA gene sequence (> 1000 nt) of strain M4 was submitted to the GenBank database under accession number OR889128. The phylogenetic tree is illustrated in Figure 1.

**FIGURE 2:**
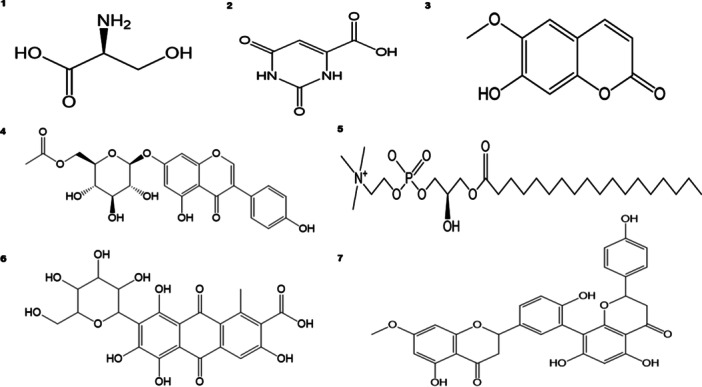
Tentative identification of the compounds in the EtOAc extract of *S. violaceorubidus* M4 using ESI‐LC‐MS.

The bioactive compounds in the ethyl acetate extract were qualitatively detected using ESI‐LCMS profiling. The extract contained eight bioactive compounds, which were categorized as amino acids, carboxylic acids, coumarins, isoflavones, phosphatidylcholine, tetrahydroxyanthraquinone, and flavonoids (Table 2 and Figure 2). The LC‐MS full‐scan chromatograms and chromatograms of the identified compounds are included in the Supporting Information.

**TABLE 2:** List of chemical constituents identified in the EtOAc extract of *S. violaceorubidus* M4 via ESI‐LC‐MS.

No.	RT (min)	MW	Tentative identification	Class	% of total
1	2.94	118.92	L‐ homoserine	Amino acid	4.14
2	2.78	156.024	Orotic acid	carboxylic acid	5.43
3	8.70	193.094	Scopoletin	Coumarin	6.72
4	9.67	474.409	6''‐O‐Acetylgenistin	isoflavone	16.51
5	10.65	523.544	Stearoyl‐sn‐glycero‐3‐phosphocholine	phosphatidylcholine	18.22
6	14.49	491.496	Carminic acid	tetrahydroxyanthraquinone	17.11
7	16.13	554.396	Biflavonoid‐flavone base + 3O and flavanone base + 2O + 1MeO	Flavonoids	19.30

*Note:* % = percent of relative concentration.

Abbreviations: RT, retention time; MW, molecular weight.

Among the tentatively identified compounds, biflavonoid‐flavone base + 3O and flavanone base + 2O + 1MeO were the most dominant, comprising 19.30% of the total compounds. This was followed by Stearoyl‐sn‐glycero‐3‐phosphocholine, which accounted for 18.22% of the total. Carminic acid ranked second, accounting for 17.11% of the total. In contrast, l‐homoserine, orotic acid, and scopoletin exhibited the lowest abundances, at 4.14%, 5.43%, and 6.72%, respectively, in the present.

### 3.2 The Extract Suppressed the Growth of Human Breast Adenocarcinoma MDA‐MB‐231 Cells

**FIGURE 3:**
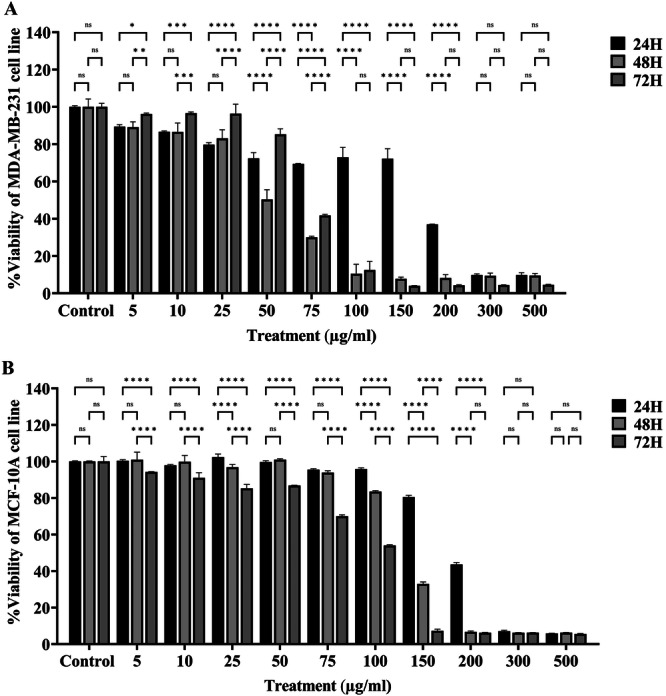
The viability of MDA‐MB‐231 (A) and MCF‐10A (B) cells after exposure to actinomycete extracts was evaluated using the MTT assay. Cells were treated with increasing concentrations of the extract (5–500 μg/mL) for 24, 48, and 72 h. The survival rate of MDA‐MB‐231 at the 48‐h time point showed an IC50 value of 48.04 μg/mL. The extract from the actinomycete culture showed specific toxicity towards MCF‐10A cells, with the IC50 values increasing to 191.5 μg/mL following a 24‐h exposure. (Control: Untreated cells). Data are represented as mean ± SEM. (****p < 0.0001, ***p = 0.0001, **p ≤ 0.001, *p < 0.05 and ns: not significant). Statistical comparisons were performed using one‐way ANOVA, followed by the LSD test. All experiments were performed in triplicate.

The cytotoxic effects of the extract on the cells were evaluated using the MTT assay. Figure 3 shows that the inhibition of cell proliferation by the extract was both time‐(24, 48, and 72 h) and dose‐dependent (0–500 μg/mL) in MDA‐MB‐231 cells. The IC50 value of the extract after 24 h was 147.5 µg/mL. At 48 h, the IC50 value for MDA‐MB‐231 cells decreased to 48.04 μg/mL, while that for MCF and MDA‐MB‐231 cells had an IC50 value of 70.08 μg/mL when the incubation period was extended to 72 h. In comparison, the extract exhibited a significantly lower cytotoxic effect on MCF‐10a cells, with high IC50 values of 191.5, 132, and 96.04 μg/mL at 24, 48, and 72 h, respectively (Figure 3).

### 3.3 Induction of Apoptosis by the Extract in the MDA‐MB‐231 Cell Line

**FIGURE 4:**
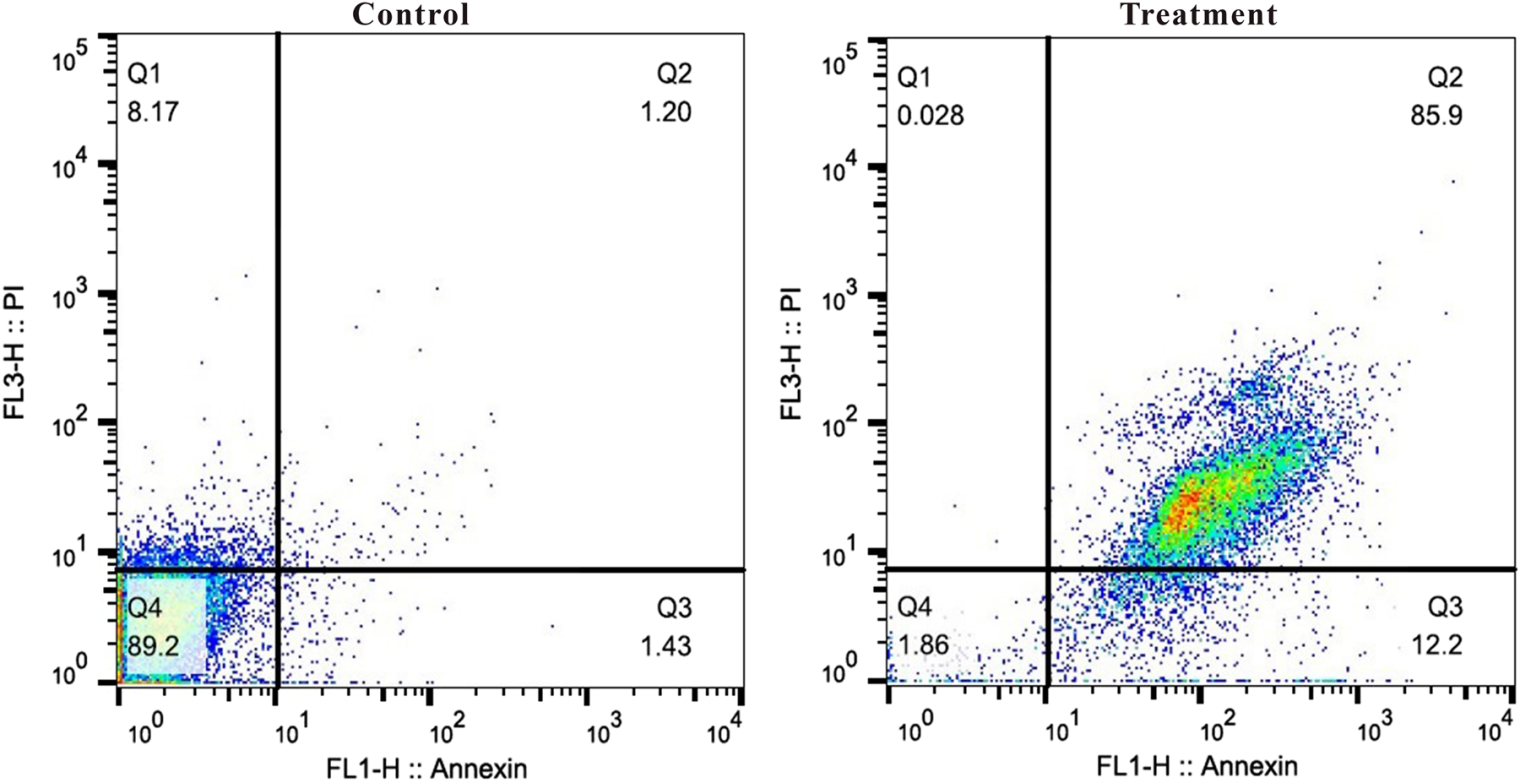
A scatter plot depicting the logarithmically amplified fluorescence signals of both viable and nonviable cells, as assessed by flow cytometry, is presented for MDA‐MB‐231 cells that were either untreated (control) or treated with 48.04 μg/mL of Streptomyces extract for 48 h (Treatment). The percentages of cells in each quadrant are shown, representing the viable (Q4), early apoptotic (Q3), late apoptotic/necrotic (Q2), and necrotic (Q1) populations. Data represent the mean of two independent experiments analyzed using FlowJo software. Statistical analysis was not performed, and the results are presented descriptively to illustrate the increase in apoptotic cell populations following treatment.

After staining with annexin V fluorescent antibody and propidium iodide, the cells were analyzed using flow cytometry. The results revealed that the extract substantially increased the proportion of apoptotic cells (98.1%) in MDA‐MB‐231 cells compared to that in untreated cells (2.63%) (Figure 4). Flow cytometric analysis demonstrated that most MDA‐MB‐231 cells treated with the extract were in the late apoptotic stage after 48 h incubation period. Minimal necrosis (0.028%) was observed in the cells during the incubation period.

### 3.4 *Streptomyces* Extract Induced Apoptosis in the MDA‐MB‐231 Cell Line Through the Extrinsic Pathway

**FIGURE 5:**
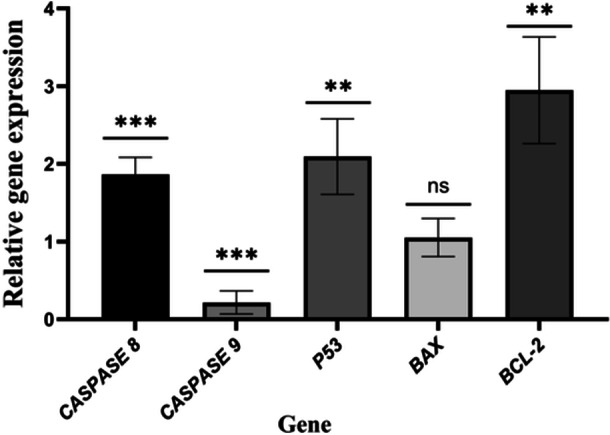
The expression levels of *BAX*, *CASPASE 8*, *CASPASE 9*, *P53*, and *BCL‐2* were analyzed using qRT‐PCR following exposure to Streptomyces extract at a concentration of 48.04 μg/mL. B2M served as a loading control (***p = 0.0001, **p ≤ 0.001, and ns, not significant).

Examining gene expression is essential for accurately determining gene function in cellular environments. We also observed reduced *CASPASE‐9* expression (0.217 ± 0.148). In the extrinsic apoptosis pathway, the death receptor gene *CASPASE‐8* was upregulated by 1.866 ± 0.218. A significant change was noted in *P53*, which exhibited a 2.094 ± 0.486‐fold increase in expression (Figure 5).

### 3.5 Antitumor Effects of *Streptomyces* Sp. M4 Extract in an Animal Model

**FIGURE 6:**
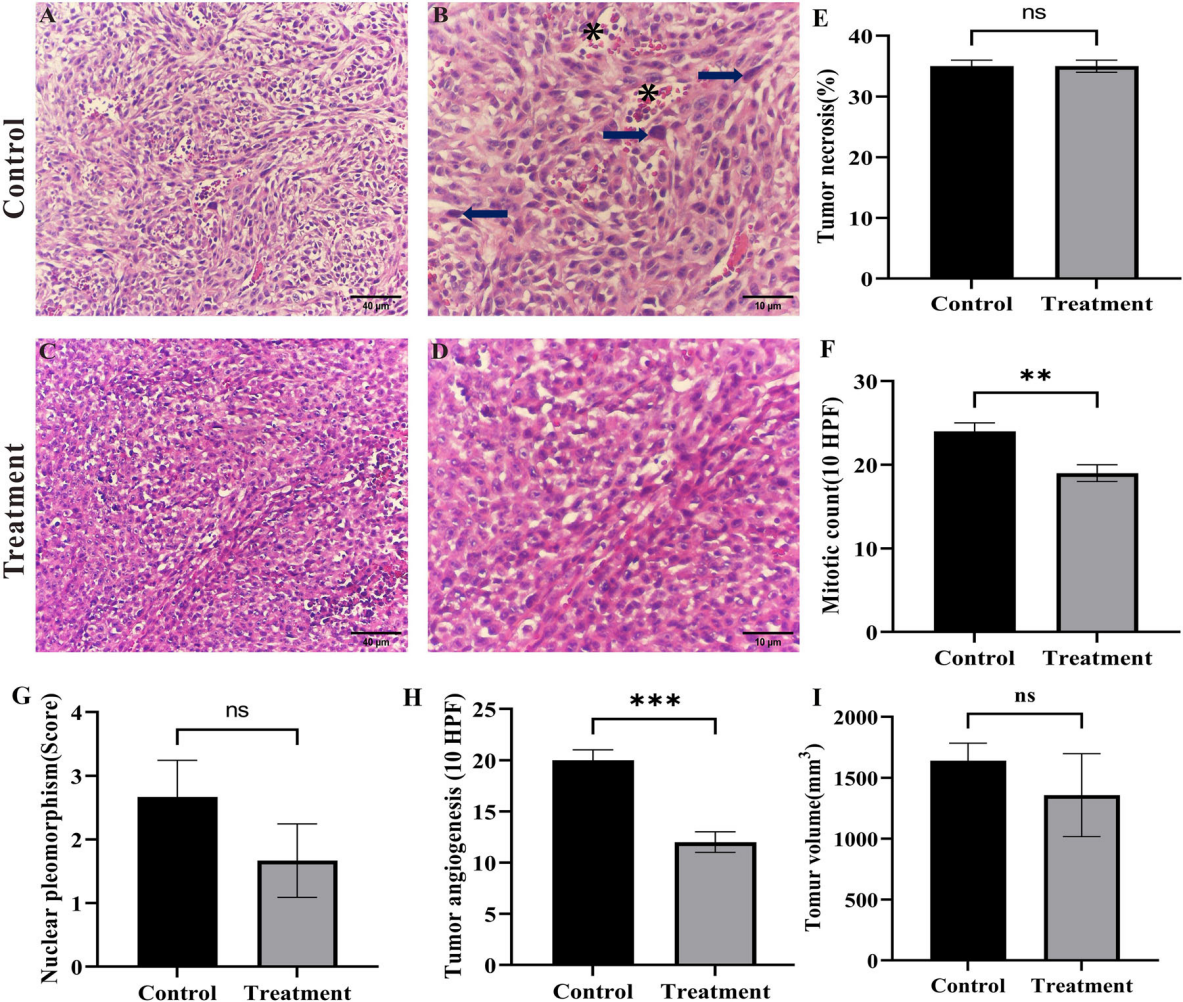
Hematoxylin and eosin (H&E) staining of breast tumor tissue sections from the control and actinomycete extract‐treated groups. (A, B) Tumor sections from the control group (C, D) and tumor sections from the treatment group. Arrows indicate nuclear pleomorphism, and asterisks indicate necrotic area. (E) Tumor necrosis (%), (F) mitotic count (per 10 high‐power fields; HPF), (G) nuclear pleomorphism score, (H) tumor angiogenesis (per 10 HPF), and (I) tumor volume were compared between the control and treatment groups. Data are presented as mean ± SEM; **p < 0.01, ***p < 0.001, ns = not significant.

The in vivo anti‐breast cancer potential of the Streptomyces extract was evaluated in mice bearing 4T1 tumors. Hematoxylin‐eosin staining analysis revealed that tumor cells in the transplanted model group were densely packed, exhibited vigorous growth, and displayed cellular atypia characterized by enlarged nuclei and distinct nucleoli. Following treatment, tumor cells in the treatment group appeared more dispersed, with visible necrotic cells present. No significant difference in tumor necrosis was observed. between the control and treatment groups, with both groups exhibiting tumor necrosis rates of 30%–40%. The mitotic count, defined as the number of mitoses per unit area, is a critical metric used in the classification and grading of tumors. Traditionally, mitotic counts are assessed by counting the number of mitoses within a high‐power field (HPF). The control group exhibited an average of 25 mitotic counts per 10 HPF, which significantly (*p* < 0.05) decreased to 19 per 10 HPF following treatment with the extract, suggesting the potential efficacy of this intervention. In the control group, nuclear pleomorphism was rated +3, whereas in the treatment group, it was reduced to +2. Additionally, tumor angiogenesis was recorded at 20 per 10 HPF in the control group, which decreased to 12 per 10 HPF after treatment with the extract. Notably, tumor‐infiltrating lymphocytes were absent in both the control and treatment groups. Regarding tumor volume, the control group exhibited an average volume of 1640.83 ± 143.08 mm³, while the treatment group showed a reduced average volume of 1358 ± 340.329 mm³. However, this reduction was not statistically significant, indicating that the extract did not significantly decrease the overall tumor burden (Figure 6).

## 4 Discussion

Halophilic actinomycetes, a distinctive group of bacteria renowned for their array of bioactive secondary metabolites, show considerable potential in the search for novel antimicrobial and anticancer therapies (Selim et al. 2021). Although actinomycetes have been the source of more than half of all discovered bioactive substances, the rate of new metabolite discovery has decreased, emphasizing the importance of investigating previously unexplored habitats. Investigations into halophilic actinomycetes have uncovered their specialized adaptations and capacity to produce vital therapeutic compounds with anticancer and anti‐inflammatory properties. Exploring these secondary metabolites is crucial to meet the pressing need for new treatments, making them invaluable assets in the fight against diseases, particularly cancer.

In this study, we investigated the effectiveness of an ethyl acetate extract obtained from a newly isolated strain that exhibited strong cytotoxic effects in the treatment. Our investigation revealed that *Streptomyces* Sp. The M4 extract effectively inhibited the proliferation of MDA‐MB‐231 cells. The inhibitory effect was both dose‐ and time‐dependent. The extract exhibited maximum efficacy at 48.04 μg/mL after a 48‐h exposure. In contrast, the extract demonstrated a markedly lower cytotoxic effect on MCF‐10a cells, which are normal mammary epithelial cells. The *Streptomyces* Sp. The M4 extract exhibited selective toxicity, killing cancer cells without harming healthy cells. This reduces drug side effects, may increase treatment effectiveness, and improve patient quality of life.

To investigate the mechanisms underlying the cell death‐inducing properties of the extract, Annexin V/PI staining was performed. The results showed a significant increase in the proportion of apoptotic cells (98.1%) in MDA‐MB‐231 cells treated with the extract compared to untreated cells, which only had a 2.63% apoptotic rate. This suggests that the extract primarily triggers cell death in TNBC cells via apoptosis rather than necrosis. To confirm this mode of action, we examined the expression levels of genes linked to both intrinsic and extrinsic apoptosis pathways. For the intrinsic pathway, we focused on *BAX*, *BCL‐2*, and *CASPASE‐9*, whereas for the extrinsic pathway, we examined *CASPASE‐8*. In addition, we analyzed the expression of *P53*, which acts as both an inhibitor of antiapoptotic genes such as *BCL‐2* and an activator of proapoptotic genes like *BAX*. The extrinsic pathway is activated by the binding of death signals to their corresponding receptors, leading to the activation of CASPASE‐8 through intracellular signaling mechanisms. The active form of *CASPASE‐8* subsequently initiates a cascade of caspase activation, although its specific role in morphological changes associated with apoptosis remains unclear (Elmore 2007). qRT‐PCR analysis indicated that *CASPASE‐8* expression was significantly increased. Following treatment with the extract. Our findings showed a reduction in *CASPASE‐9* expression, with no involvement of the intrinsic apoptosis pathway in the anticancer properties of the extract. Additionally, we examined the changes in *P53* expression in MDA‐MB‐231 cancer cells after treatment with the extract. The results revealed that *p53* expression was higher in the treated cells than in the control group. This finding suggests that the extract predominantly induces apoptosis via the extrinsic pathway. Consistently, Elmallah et al. showed that compounds such as sharkquinone, resistomycin, undecyl prodigiosin, butyl cyclopentyl prodigiosin, elloxizanone A and B, carboxyexfoliazone, and exfoliazone derived from actinomycetes isolated from the Egyptian Red Sea were capable of inducing apoptosis in MDA‐MB‐231 cells (Elmallah et al. 2020).

Tumor necrosis and nuclear pleomorphism are frequently linked to the aggressive progression of tumors and the occurrence of metastasis and are considered prognostic indicators of unfavorable outcomes in patients diagnosed with breast, lung, and kidney cancers (Liu and Jiao 2020; Singh and Lele 2022). The study found no notable difference in the occurrence of tumor necrosis between the control and treatment groups of mice in vivo, as both groups exhibited tumor necrosis rates of 30%–40%. In the control group, nuclear pleomorphism was scored as +3, whereas in the treatment group, it decreased to +2. This reduction indicates a lower degree of nuclear atypia, with more uniform nuclear sizes and shapes and less pronounced malignant features. Nuclear pleomorphism is one of the three major components of the Nottingham histological grading system, along with mitotic count and tubule formation (Elston and Ellis 1991). A lower pleomorphism score contributes to a lower overall histological grade, which is strongly correlated with less aggressive tumor behavior and better prognosis in patients with breast cancer (van Dooijeweert et al. 2022; Galea et al. 1992; Rakha et al. 2008). From a therapeutic perspective, the ability of our extract to reduce nuclear pleomorphism suggests that it may suppress malignant transformation, attenuate tumor progression, and potentially improve clinical outcomes. Tumor angiogenesis was 20 per 10 HPF in the control group and reduced to 2 per 10 HPF after treatment with the extract. These findings from the TNBC animal model confirmed that the extract not only inhibited growth and induced cell death in TNBC cells but also exerted significant antitumor effects in vivo, inhibiting tissue processes associated with cancer growth and metastasis.

Overall, the findings from both in vitro and in vivo experiments consistently demonstrate the antitumor potential of the extract. In the in vitro setting, treatment of MDA‐MB‐231 cells with the extract resulted in a pronounced induction of apoptosis, with more than 98% of cells undergoing apoptotic death, predominantly in the late apoptotic phase, whereas necrosis remained negligible. Complementary in vivo analyses revealed that, although no significant difference was observed in tumor necrosis between the control and treated groups, a marked reduction in mitotic activity was evident. Specifically, the mitotic index significantly decreased from 25 to 19 mitoses HPF following extract administration. These results suggest that the antitumor efficacy of the extract is primarily mediated through the induction of apoptosis and suppression of cellular proliferation rather than through necrotic mechanisms. LC‐MS analysis of the *S. violaceorubidus* M4 extract identified eight compounds as major metabolites, several of which have been studied for their anticancer effects in previous research. Balhouse et al. investigated the effects of N‐(3‐oxododecanoyl)‐l‐homoserine lactone (OdDHL) on breast cancer cells (MDA‐MB‐231 and MCF‐7) and normal breast cells (MCF‐10A). They discovered that OdDHL significantly reduced the viability of MDA‐MB‐231 cells, moderately affected MCF7 cells, and had no effect on MCF‐10A cells (Balhouse et al. 2017). Orotic acid (2) has been shown to have anticancer effects. This effect was unrelated to changes in apoptosis‐related gene expression but was linked to enhanced caspase‐3/7 activity (Marynowicz et al. 2023). Scopoletin has previously been shown to have anti‐breast cancer properties (Yu et al. 2021). This compound exhibited enhanced antiproliferative effects on MCF‐7 and MDA‐MB‐231 cell lines while exhibiting reduced toxicity towards MCF‐10A breast epithelial cells. Studies on its pharmacological mechanisms have revealed that scopoletin halts the cell cycle at the G2/M phase, diminishes PI3K and Akt phosphorylation in MCF‐7 cells, and modulates apoptotic protein expression to induce cell death. Furthermore, it was found to suppress MCF‐7 cell growth in an animal model (Yu et al. 2021). 6''‐O‐Acetylgenistin, Stearoyl‐sn‐glycero‐3‐phosphocholine, Carminic acid, and biflavonoid‐flavone base + 3 O and flavanone base + 2 O + 1MeO were additional compounds identified in this extract, with previous studies confirming their anticancer effects in various cell lines and models (Spagnuolo et al. 2015; Naeem et al. 2023; Konstantinou et al. 2024; Lima et al. 2024). Current TNBC therapies rely heavily on chemotherapeutics, such as doxorubicin and mitomycin C, both derived from actinomycetes (Shuhendler et al. 2007; Moradi Gardeshi et al. 2022), which are effective but constrained by severe toxicities. Doxorubicin is associated with cumulative dose‐dependent cardiotoxicity (Shrestha et al. 2023; Goje et al. 2025). Although clinically valuable, mitomycin C carries the risk of hemolytic–uremic syndrome and pulmonary toxicity (Verweij et al. 1987). In contrast, M4 extract selectively induced apoptosis via the extrinsic pathway and reduced angiogenesis, indicating a therapeutic profile that could minimize systemic side effects compared to conventional agents.

Moreover, the established clinical success of actinomycete‐derived drugs (e.g., doxorubicin, epirubicin, and bleomycin) underscores the feasibility of developing halophilic actinomycete metabolites as new anticancer agents (Rui et al. 2025). The presence of apoptosis‐modulating compounds, such as scopoletin and orotic acid, in the M4 extract reinforces its potential for further translational development.

In future studies, it will be essential to focus on compound isolation, structural elucidation, pharmacokinetic profiling, and combinatorial testing with established TNBC drugs to explore their synergistic effects. These investigations are critical for confirming the specific molecules responsible for the observed effects and defining their therapeutic indices. Addressing these limitations will strengthen the translational potential of the M4 extract and pave the way for its advancement toward preclinical and clinical evaluations.

## 5 Conclusion

This study emphasizes the toxic impact of *Streptomyces violaceorubidus* M4 extract on TNBC. The findings demonstrated cytotoxicity and apoptosis induction in a cellular model, which was further validated by in vivo analyses. Administration of the extract to tumor‐bearing animals significantly reduced nuclear pleomorphism and the number of mitotic cells in the tumor tissue. These results suggest that the extract derived from this strain represents a valuable source of pharmaceutical metabolites for anticancer applications.

## Author Contributions

A.Z. conducted the experiments and drafted the main text. E.S. contributed to the manuscript writing, performed editing, and prepared the supplementary materials. H.J. and M.T. were responsible for manuscript editing. All authors have reviewed the final manuscript. A.Z. conducted the experiments and drafted the main text. E.S. contributed to the manuscript writing, performed editing, and prepared the supplementary materials. H.J. and M.T. were responsible for manuscript editing. All authors have reviewed the final manuscript.

## Conflicts of Interest

The authors declare no conflicts of interest.

## Supplementary Data

https://drive.google.com/drive/folders/1PoRH1CkeO7E2UTTiTsKniw2286xMvARa.

## Depository Data

https://drive.google.com/drive/folders/1NcHXVK5OFmd00bLJHrJRJG0J1CniDHR4.

## Supporting information

Supp information.

Supp information.

Supp information.

## Data Availability

Data underlying this article are publicly available in the Gene Bank database and can be accessed with OR889128. Other Data are publicly available in the figshare repository as part of these records: https://docs.google.com/document/d/1VN6fAQnjK8f1qwNmO36TS89Y6_Q7Sto8/edit?usp=drive_link&ouid=114469081100773289223&rtpof=true&sd=true.
