# Supplementary figures and images for: Antitumor Compounds From Halophilic Streptomyces violaceorubidus M4 Against Triple‐Negative Breast Cancer
Source: Microbiologyopen. 2025 Oct 27;14(6):e70095. doi: 10.1002/mbo3.70095 (PMC12558596; doi:10.1002/mbo3.70095)

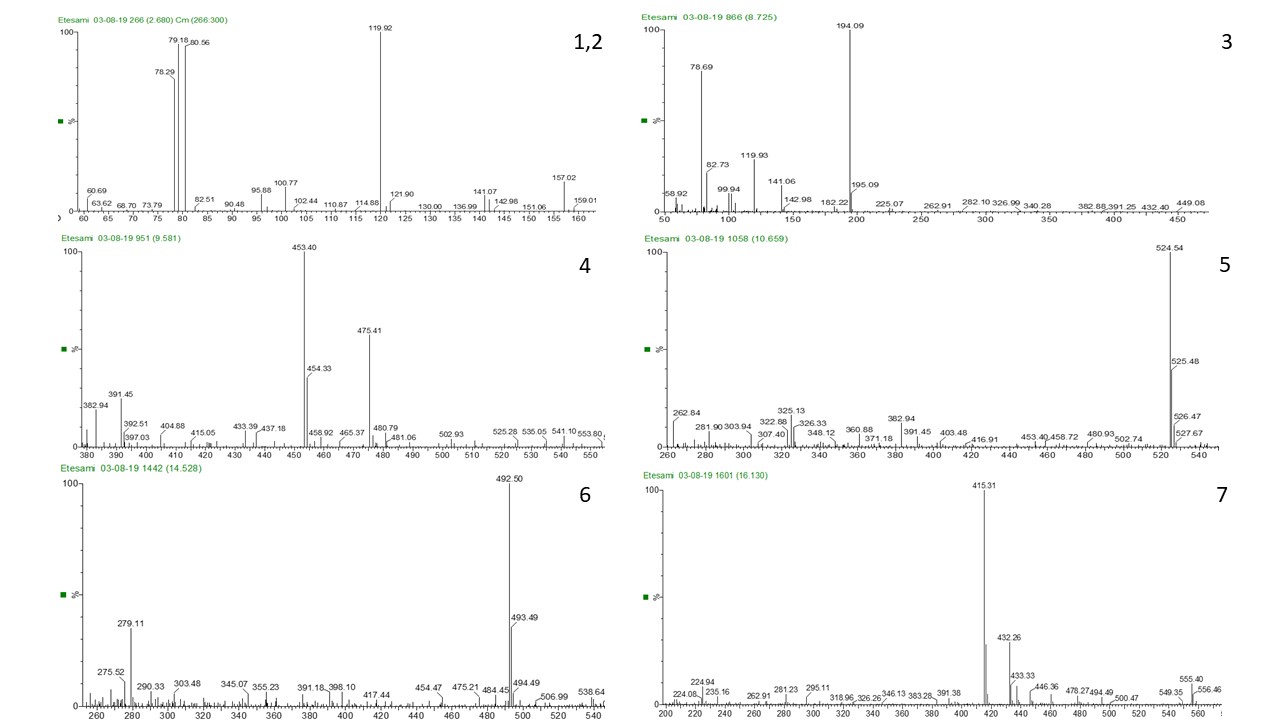

Supplement: Supplementary file 1 — Supp information. [file MBO3-14-e70095-s001.jpg]

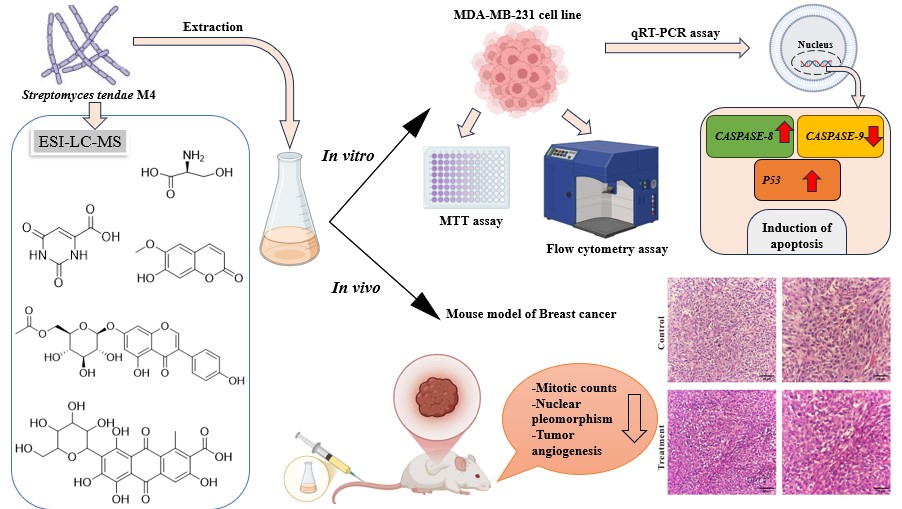

Supplement: Supplementary file 2 — Supp information. [file MBO3-14-e70095-s002.jpg]

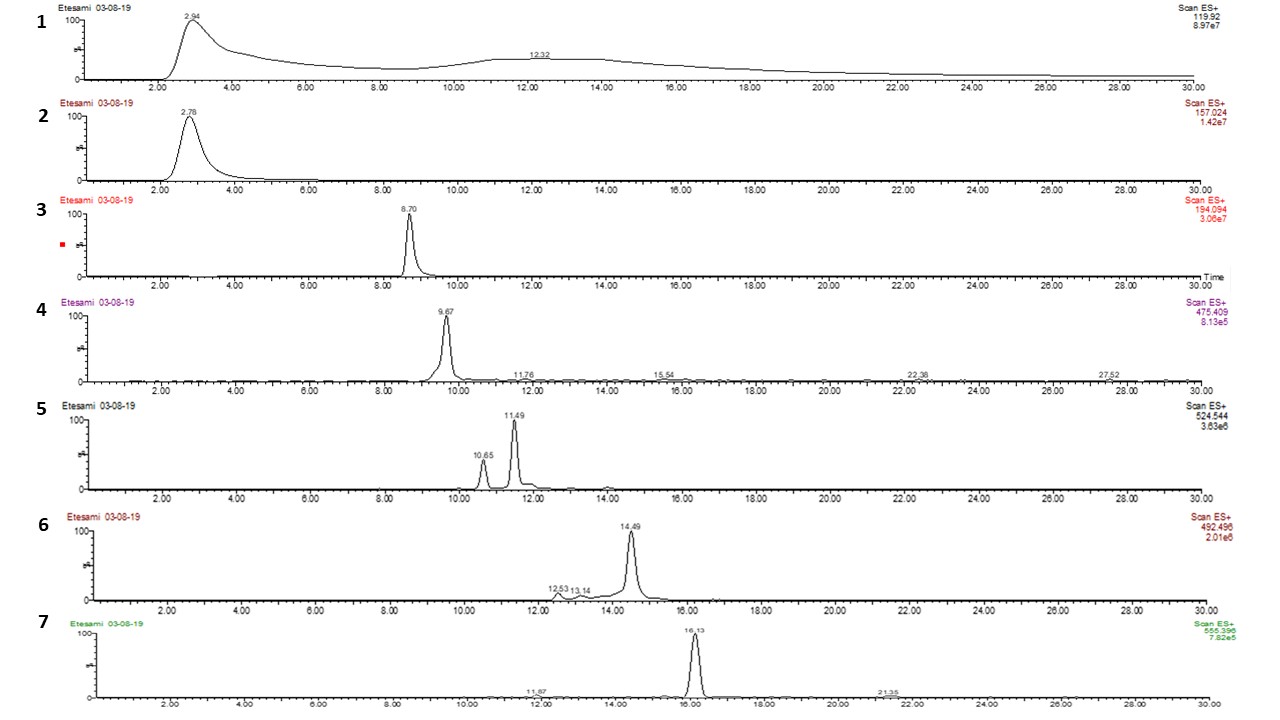

Supplement: Supplementary file 3 — Supp information. [file MBO3-14-e70095-s003.jpg]
